# Lactate Chemical Exchange Saturation Transfer (LATEST) Imaging *in vivo* A Biomarker for LDH Activity

**DOI:** 10.1038/srep19517

**Published:** 2016-01-22

**Authors:** Catherine DeBrosse, Ravi Prakash Reddy Nanga, Puneet Bagga, Kavindra Nath, Mohammad Haris, Francesco Marincola, Mitchell D. Schnall, Hari Hariharan, Ravinder Reddy

**Affiliations:** 1 Center for Magnetic Resonance and Optical Imaging, Department of Radiology, Perelman School of Medicine, University of Pennsylvania, Philadelphia PA; 2 Laboratory of Molecular Imaging, Department of Radiology, Perelman School of Medicine, University of Pennsylvania, Philadelphia PA; 3 Research Branch, Sidra Medical and Research Center, Doha, Qatar

## Abstract

Non-invasive imaging of lactate is of enormous significance in cancer and metabolic disorders where glycolysis dominates. Here, for the first time, we describe a chemical exchange saturation transfer (CEST) magnetic resonance imaging (MRI) method (LATEST), based on the exchange between lactate hydroxyl proton and bulk water protons to image lactate with high spatial resolution. We demonstrate the feasibility of imaging lactate with LATEST in lactate phantoms under physiological conditions, in a mouse model of lymphoma tumors, and in skeletal muscle of healthy human subjects pre- and post-exercise. The method is validated by measuring LATEST changes in lymphoma tumors pre- and post-infusion of pyruvate and correlating them with lactate determined from multiple quantum filtered proton magnetic resonance spectroscopy (SEL-MQC ^1^H-MRS). Similarly, dynamic LATEST changes in exercising human skeletal muscle are correlated with lactate determined from SEL-MQC ^1^H-MRS. The LATEST method does not involve injection of radioactive isotopes or labeled metabolites. It has over two orders of magnitude higher sensitivity compared to conventional ^1^H-MRS. It is anticipated that this technique will have a wide range of applications including diagnosis and evaluation of therapeutic response of cancer, diabetes, cardiac, and musculoskeletal diseases. The advantages of LATEST over existing methods and its potential challenges are discussed.

Changes in lactate metabolism are associated with a wide variety of diseases, including cancer^1^, cardiac failure^2^, liver disease^3^, diabetes mellitus^4^, and neurologic disorders such as epilepsy^5^. As a result of the Warburg effect, tumors exhibit up-regulated lactate dehydrogenase (LDH), leading to increased levels of lactate^6^. Many studies have shown that tumor lactate levels correlate with increased metastasis, tumor recurrence, and poor outcome^7^,^8^. Lactate also plays a role in promoting tumor inflammation and can function as a signaling molecule that stimulates tumor angiogenesis^9^. Thus, non-invasive measurement of lactate is of tremendous significance to the study of metabolic defects in a wide range of pathologies.

Currently there are two major methods employed in measuring lactate *in vivo*. One is traditional magnetic resonance spectroscopy (MRS; both ^1^H and ^13^C)^10^,^11^, which has been used to measure both static lactate levels and dynamic changes. However, these are limited by inadequate sensitivity and spatial resolution. The other method involves ^13^C-labeled pyruvate infusion and dynamic nuclear polarization (DNP), which provides greater than 10,000 fold signal enhancement compared to conventional MRS^12^,^13^,^14^. Despite its high sensitivity, this method only probes fast kinetics (<1 min) of lactate turnover from ^13^C-labeled pyruvate and it requires special equipment and complex modeling for data analysis.

Here, we describe a magnetic resonance imaging (MRI) method based on **la**c**t**ate chemical exchange saturation transfer (C**EST**) to image lactate (**LATEST**). In this work, we use the CEST technique^15^,^16^,^17^ to exploit the exchange of –OH protons on lactate with bulk water. CEST has been previously utilized to image amine protons of metabolites such as glutamate^18^ and guanidine protons of creatine^19^, as well as amide and hydroxyl protons of proteins^20^,^21^ and hydroxyl protons from metabolites such as glucose^22^, glycogen^23^, myoinositol^24^, and glycosaminoglycans^25^. This method utilizes standard proton MRI and requires neither ^13^C labeled pyruvate nor DNP polarization. Thus far, no studies have used CEST to image the hydroxyl protons of lactate *in vivo*. First, we examine the pH and concentration dependence of LATEST in phantoms. Then, the feasibility of measuring LATEST *in vivo* is demonstrated in a lymphoma tumor model, and in human skeletal muscle. Dynamic changes in LATEST are reported in tumors pre- and post-infusion of pyruvate, and in exercising human skeletal muscle. LATEST measurements are compared to lactate measured with multiple quantum filtered proton magnetic resonance spectroscopy (SEL-MQC ^1^H-MRS)^26^. The advantages and challenges to LATEST are discussed.

## Results

### Phantom studies

**Figure 1:**
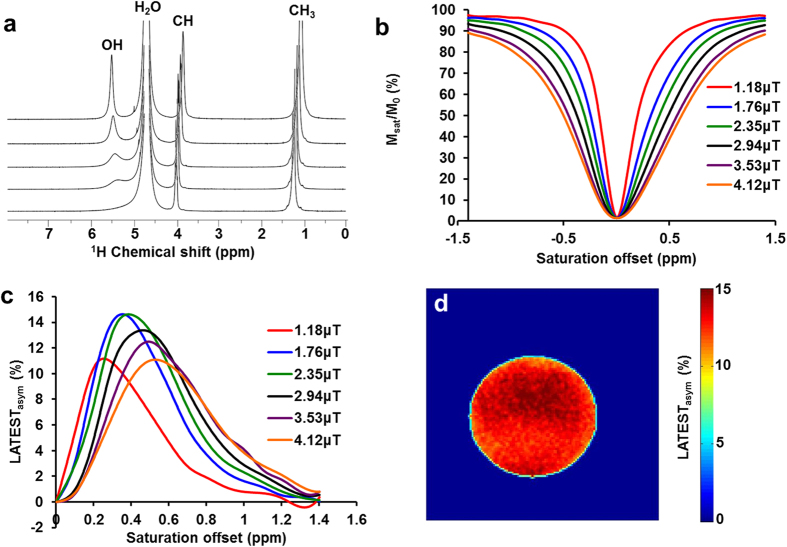
Determination of lactate –OH resonance. (**a**) High-resolution 1D-^1^H NMR spectra of 15M sodium lactate (in PBS buffer at pH 7.1 ± 0.1) at varying temperatures acquired with Bruker Avance DMX 400 MHz spectrometer equipped with a 5 mm PABBI proton probe, (**b**) Z-spectrum and (**c**) asymmetry plot for 50mM lactate at 9.4T, pH 7, at 37ºC with B1_rms_ titrated from 1.18 µT - 4.12 µT, with a saturation duration of 5s, (**d**) CEST map for 50mM lactate at 9.4T at 0.4ppm with 2.35 µT and 5 s duration.

**Figure 2:**
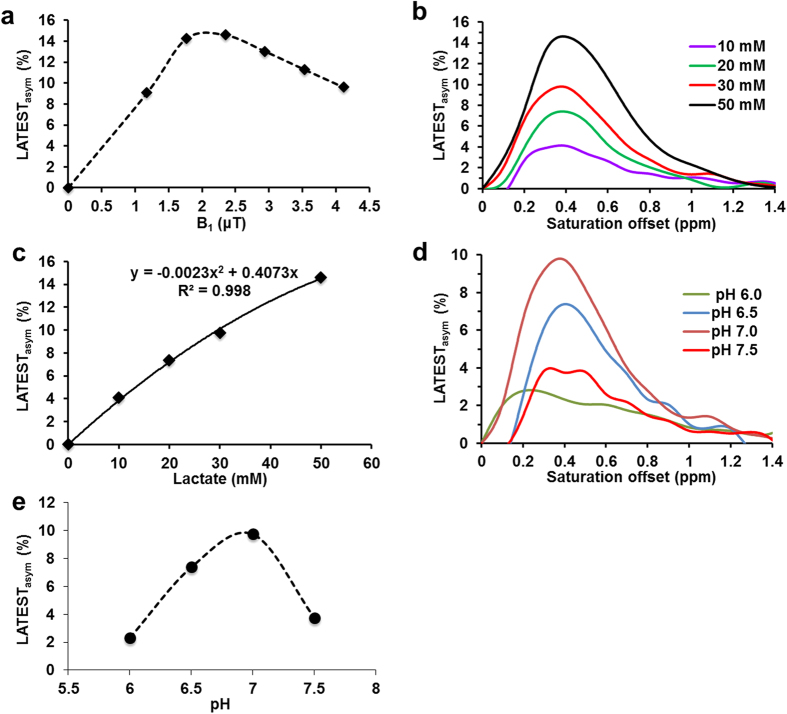
Characterization of LATEST. (**a**) LATEST dependence on B_1_ from a 50 mM lactate phantom at pH 7, at the saturation duration of 0.4 ppm offset from water, (**b**) asymmetry plot of LATEST at 10, 20, 30, and 50 mM lactate at pH 7, with B_1rms_ = 2.35 μT and pulse duration = 5 s, (**c**) concentration dependence of LATEST at 0.4ppm, pH 7, with B_1rms_ = 2.35 μT and pulse duration = 5 s, (**d**) asymmetry plots from 30 mM lactate, pH = 6, 6.5, 7, and 7.5 (**e**) pH dependence of LATEST at 0.4 ppm from 30 mM lactate with B_1rms_ = 2.35 μT, duration = 5 s. All data acquired at 37 °C, 9.4 T.

The chemical shift of the hydroxyl (–OH) proton resonance of sodium lactate, measured by 1D ^1^H NMR, varies from ~0.8 to 0.4 ppm offset from water as the temperature is changed from 4 °C to 27 °C. At 37 °C, the –OH resonance is not clearly visible by ^1^H NMR, owing to the significant exchange broadening and proximity to the water resonance (Fig. 1a). Consequently, the z-spectrum of 50 mM sodium lactate obtained at 9.4 T does not exhibit any sharp features at 37 °C (Fig. 1b). However, the CEST asymmetry plot of the same exhibits clear resonance centered between ~0.3 to 0.5 ppm (Fig. 1c). Typically, this peak is masked in the z-spectrum by the overwhelming water signal. In the asymmetry plot, the subtraction of the water signal elucidates the lactate –OH resonance at 0.4ppm. A representative CEST map at 0.4ppm of the 50 mM lactate phantom is shown in Fig. 1d. Optimal LATEST parameters in phantoms at 9.4 T were B_1rms_ = 2.35 μT, with a 5 s duration (Fig. 2a). With the imaging parameters described, at neutral pH and 37 °C, lactate exhibits ~0.4% CEST/mM at 9.4 T (Fig. 2b,c). The pH dependence of 30 mM lactate at 9.4 T shows maximum LATEST signal at pH = 7 (~10% LATEST asymmetry at 0.4ppm downfield from water) (Fig. 2d,e). At both lower and higher pH, a decrease in LATEST asymmetry at 0.4ppm is observed.

At 7 T, the optimal parameters for LATEST in phantoms are: 5 s saturation length with ~1.09 μT B_1rms_ at 25 °C and 1.46 μT B_1rms_ at 37 °C. With the imaging parameters described, at neutral pH and 25 °C, lactate exhibits 0.25% CEST/mM at 7 T, with 1.09 μT (B_1rms_). Based on the experimental signal-to-noise ratio (SNR), this method has sufficient sensitivity to detect 2 to 3 mM lactate.

The exchange rate (k) estimated from lactate phantoms (pH 7) at 25 °C is ~350 ± 50 s^−1^ and at 37 °C is ~550 ± 50 s^−1^. Therefore, the lactate chemical exchange rate is in the slow to intermediate condition and meets the requirement for observing the CEST effect for field strengths greater than 4 T.

### Animal model studies: LATEST imaging of lymphoma flank tumors

**Figure 3:**
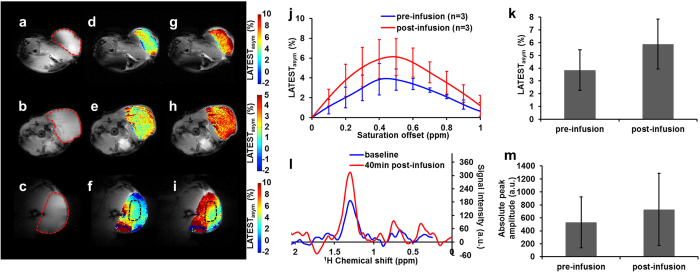
LATEST from lymphoma tumors. (**a–c**) Anatomical image from three animals, with flank tumor region indicated by dotted red line, and the LATEST maps (**d–f**) pre-infusion and (**g–i**) post-infusion with (**j**) corresponding asymmetry plots (asymmetry from Animal 3 in the third row is taken from region indicated in dotted black line), (**k**) LATEST change at 0.4ppm from three animals pre- and post-infusion, (**l**) representative SEL-MQC^1^H-MRS pre- and 40 minutes post-infusion from flank tumor showing (**m**) increase in lactate peak amplitude from three animals (~40%) from spectroscopy.

Anatomical images of flank tumors on three mice are shown in Fig. 3a–c. Baseline CEST maps from the tumor regions of each animal (Fig. 3d–f) show an average LATEST_asym_ of ~3.5%. Following infusion of 300 mM pyruvate through the tail vein, the LATEST signal increased in the tumor regions (Fig. 3g–i). Average asymmetry plots from the tumor regions (Fig. 3j) showed an endogenous LATEST peak and subsequent increase post-infusion, centered ~0.5ppm downfield from water. The asymmetry plot from one animal (row 3 of Fig. 3a–i) was obtained from the region of interest (ROI) indicated in the black dotted line (Fig. 3f). This region was used in order to avoid regions with large B_0_ inhomogeneity, which was observed in the outer region of the tumor (Supplementary Fig. 1). Data from lymphoma tumors of three animals showed a ~60% increase in LATEST asymmetry after ~40 minutes post-infusion of pyruvate (Fig. 3k).

In tumors, endogenous lactate levels are expected to be in the range of 2 to 10 mM^27^. Baseline LATEST observed in the tumor model is largely due to endogenous lactate, based on the ~0.4% LATEST asymmetry per mM of lactate observed in phantoms at 9.4 T.

Tumor lactate was also measured in three animals with flank tumors, using SEL-MQC ^1^H MRS. Spectroscopy results pre- and post-infusion of pyruvate are shown for a representative animal (Fig. 3l). The increase in lactate peak amplitude after pyruvate infusion shown by spectroscopy (Fig. 3m) from three animals shows a trend in lactate change that is similar to the trend observed with LATEST.

### Human studies: LATEST imaging of healthy human calf muscle

**Figure 4:**
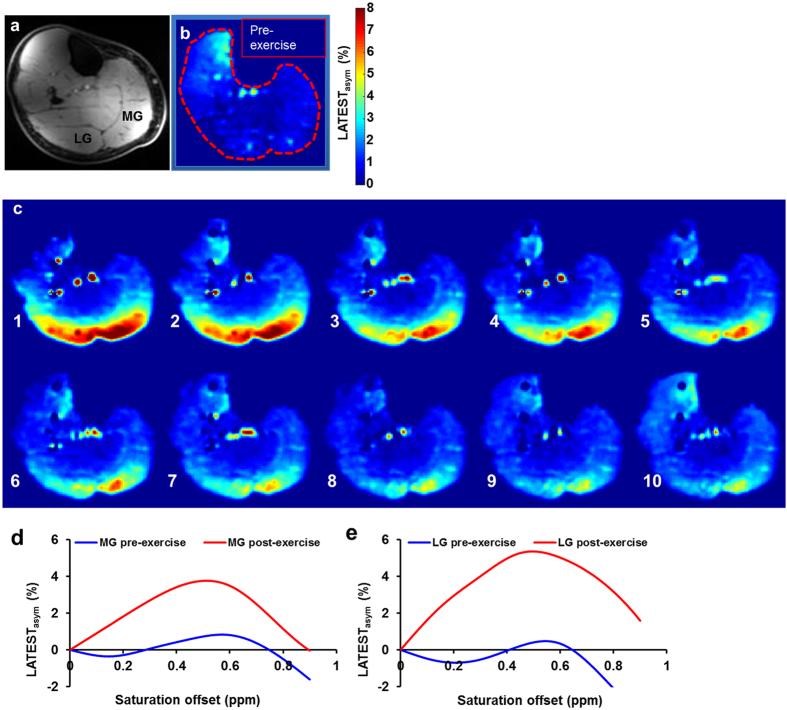
*In vivo* LATEST. (**a**) anatomical image of human calf muscle, (**b**) two pre-exercise, resting-state CEST maps showing ~1% LATEST_asym_ at 0.5ppm, and (**c**) after 3 minutes of exhaustive exercise, ten post-exercise images acquired over 18 minutes: the first image (c1) obtained 3 minutes after cessation of exercise shows a CEST asymmetry increase in the medial gastrocnemius (MG) and lateral gastrocnemius (LG) muscles of ~4–6%. Each of the subsequent images (c2–10), acquired with a resolution of 1.8 minutes, shows lactate recovery in the MG and LG. All LATEST images acquired using B_1rms_ = 0.73 μT and 3 s duration; Asymmetry plots, corrected for B_0_ and B_1_, for pre- and post-exercise LATEST of the (**d**) medial gastrocnemius (MG) and (**e**) lateral gastrocnemius muscles from a representative subject, acquired with B_1rms_ = 0.73 μT, 3 s duration.

**Figure 5:**
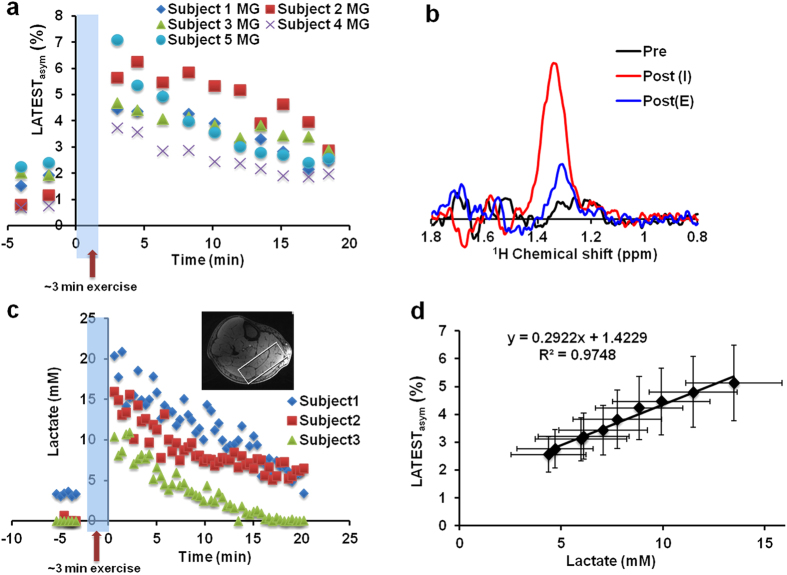
*In vivo* LATEST and SEL-MQC spectroscopy. (**a**) LATEST at 0.5ppm in the medial gastrocnemius (MG) of resting-state calf muscle, and post-exercise recovery from 5 healthy volunteers, (**b**) representative lactate MRS in a voxel from the MG/LG pre-exercise, immediately post-exercise, and after 20 minutes of recovery, and (**c**) pre- and post-exercise lactate edited MRS data from 3 healthy volunteers (representative voxel location from one subject shown in insert), (**d**) correlation of lactate concentration from spectroscopy and LATEST from the MG. Error bars indicate standard error.

Healthy human calf muscle (Fig. 4a) exhibited a resting-state LATEST asymmetry of about 1.5% (Fig. 4b). This is consistent with the reported concentration of endogenous lactate (3.8 ± 1.1 mM) in muscle under resting conditions^28^. It also indicates that, with the experimental parameters used, contributions from any other endogenous metabolites to LATEST are small. However, in the first LATEST image, acquired 3 minutes after cessation of exercise, LATEST asymmetry increased in exercising muscle (gastrocnemius muscle, activated through plantar flexion) to ~4–7%, which recovered to baseline over period of 20 minutes (Fig. 4c). The asymmetry plots from the medial and lateral gastrocnemius muscles from the same subject pre-exercise, and immediately post-exercise, are shown in Fig. 4d,e. Similar increase in post-exercise LATEST is consistently observed in five healthy volunteers (Fig. 5a). Lactate concentration derived from SEL-MQC based edited spectra (Fig. 5b) pre- and post-exercise from 3 healthy volunteers exhibits the same trend (Fig. 5c) as the LATEST. The LATEST correlates well (R^2^ = 0.97) with an intercept that indicates lactate concentration from SEL-MQC spectroscopy is under-estimated (Fig. 5d). Based on the slope value of ~0.29% per mM of lactate from spectroscopy, we estimate post-exercise muscle lactate levels to be approximately 14–25 mM. These results are consistent with reported lactate concentration increase of ~20 mM measured in muscle biopsy after intense exercise^29^.

*In vivo* CEST images, both from human skeletal muscle and from animal tumors, were corrected for B_0_ and B_1_ inhomogeneity (Supplementary Fig. 1). We have also included z-spectra for skeletal muscle and animal tumors in Supplementary Fig. 2.

Following intense exercise, the muscle T_2_ is expected to change, which may confound the LATEST results. To address this issue, we computed T_2_ maps of skeletal muscle under identical exercising conditions and found that T_2_ is elevated by <10% immediately after exercise, and stayed constant over 20 minutes (Supplementary Fig. 3). We estimated that this very small change in the T_2_ would have a negligible contribution to LATEST.

## Discussion

The results presented on phantoms, tumor model, and skeletal muscle demonstrate the feasibility of lactate measurement with the LATEST technique. Based on the results of the phantom experiments, we have chosen B_1_ parameters that provide optimal CEST effect at 0.4ppm.

The application of this method in tumors was demonstrated in Fig. 3 in a lymphoma mouse model. As discussed in the introduction, elevated tumor lactate levels contribute to cancer progression and are correlated with poor patient outcome. The enzyme responsible for lactate production, LDH, has become a target for cancer therapy. It catalyzes the inter-conversion of pyruvate and lactate with simultaneous conversion of NADH and NAD^+^. In tumors, when oxygen is absent or in short supply (hypoxia), LDH converts pyruvate to lactate^6^. Even in the presence of sufficient oxygen, tumor cells derive their energy from glycolysis (Warburg effect) leading to increased production of lactic acid^11^,^30^,^31^,^32^. With LATEST, we can investigate tumor glycolysis through injection of non-enriched pyruvate or glucose^33^,^34^.

For the mouse tumor studies, due to long acquisition times, we have acquired LATEST only at one time point following the infusion of pyruvate solution in tumors. However, with further optimization of the protocol it is feasible to gather time dependent data post-infusion. Although we have demonstrated the LATEST measurement pre- and post- pyruvate infusion, similar studies can be performed using glucose infusion. Previous studies with GlucoCEST have shown that there is a measurable GlucoCEST contrast in tumors after glucose infusion, which has been attributed primarily to the extracellular glucose^22^,^35^. Thus, LATEST and GlucoCEST are expected to provide complementary information from tumors.

When developing new CEST biomarkers, it is necessary to consider other possible contributions to the CEST signal. Due to the presence of –OH groups from other endogenous molecules (glucose, glycogen, etc.) it is possible that the baseline, endogenous LATEST signal may have contributions from these molecules. However, as shown previously, −OH groups from glucose and glycogen have resonances at around 1 ppm as opposed to ~0.4ppm in the case of lactate^22^,^23^. Other metabolites that may be present in tumors, such as pyruvate, do not have exchangeable hydroxyl protons, and would not be expected to contribute to the LATEST signal at 0.4ppm. In skeletal muscle, we must consider the possible contribution of creatine to the LATEST signal. Creatine CEST (CrCEST) experiments by Kogan *et al.*^19^ demonstrated that the guanidine protons of creatine resonate farther downfield at 1.8ppm from bulk water, and require a much shorter saturation pulse (500 ms) and higher B_1_ power of 2.9 μT. Furthermore, if creatine contributed to the LATEST effect at ~0.5ppm, we would have observed higher signal in the resting state skeletal muscle. Additionally, increased post-exercise creatine has been shown to recover within ~2 minutes, which is much shorter than the ~18 minutes it takes for the LATEST signal to dissipate post-exercise. Based on *in vivo* asymmetry plots presented in Fig. 4 and the intercept in Fig. 5d, the contributions to LATEST from other metabolites are small with the given saturation parameters. The primary use of the LATEST method is in the dynamic measurement of changes in lactate, like in pyruvate or glucose infusion in tumor, post-exercising muscle experiments.

To the best of our knowledge, there is no previously reported CEST method that exploits –OH groups of lactate for imaging lactate activity in tumor and other pathologies/tissues. The major advantage of this method is that it is not affected by lipid resonances that swamp ^1^H-MRS- derived lactate. In addition, this method has a sensitivity enhancement of two orders of magnitude compared to conventional ^1^H-MRS-based detection of lactate. Considering the sensitivity differences in ^13^C and ^1^H, LATEST sensitivity is comparable to DNP. We have demonstrated the feasibility of LATEST *in vivo* at both 9.4 T and 7 T.

While there are clear advantages of LATEST imaging compared to other methods, there are also some challenges to address. Because the hydroxyl protons of lactate resonate so close to the large water peak, there is a greater effect of direct saturation. A robust B_0_ correction method is required for LATEST (see Supplementary Methods). In this work, the images were corrected for B_0_ and B_1_ inhomogeneities prior to computation of CEST asymmetry (Supplementary Fig. 1).

In summary, with studies on phantoms, animal models of tumors, and in exercising human skeletal muscle, we demonstrated that the LATEST method enables high-resolution imaging of lactate *in vivo*, without expensive contrast agents. In terms of sensitivity, the LATEST method out-performs conventional ^1^H-MRS. Given the increasing availability of 7 T MRI scanners, the LATEST method can be readily translated into clinical settings to study patients with cancer and other metabolic diseases. Potential applications of the method include: tumors diagnosis, probing tumor glycolytic activity, and monitoring of response to therapies in cancer and a variety of metabolic disorders.

## Methods

High-resolution 1-D ^1^H NMR phantom experiments were performed on a vertical bore Bruker Avance DMX 400 MHz spectrometer (Bruker Corporation, Germany), equipped with a 5 mm PABBI proton probe. Phantom experiments and animal experiments were performed on a 9.4 T, 30 cm horizontal bore magnet (Agilent, USA) interfaced to a Varian console, with a 20 mm volume coil (M2M Imaging, USA), and a custom-built surface coil/loop coil.

Human skeletal muscle studies and additional phantom studies were performed on 7 T whole-body MRI scanner (Siemens, Erlangen, Germany) with a 28-channel QED knee RF coil.

### Phantom Preparations

For the high-resolution NMR experiments, 15M sodium lactate was prepared in PBS buffer at pH = 7.1.

For the concentration dependent study at 9.4 T, phantoms with 10, 20, 30 and 50 mM sodium lactate (Sigma Aldrich, USA) concentration in phosphate buffered saline (PBS) were prepared in 15 mm glass tubes at pH 7.0. For the pH-dependent study, 30 mM lactate samples were initially prepared in PBS at pH 7, and pH was adjusted to 6.0, and 6.5 using 1N HCl, and adjusted to pH 7.5 with NaOH. The temperature was maintained at 37 °C by blowing a warm air directed at the tube and monitored using a thermocouple attached to the NMR tube.

### Phantom Imaging: 9.4 T

For the 15M sodium lactate sample imaging at 9.4 T (vertical bore), a sealed capillary containing a mixture of D_2_O (for lock) and 10 mM tetramethylsilane (TMS) (for reference) was inserted into the tubes. The spectral parameters used were 2 dummy acquisitions followed by 24 acquisitions (pulse sequence: ‘zz30.sw’), 65536 TD (real+imaginary), 6775 Hz sweep width, and a relaxation delay of 4 s. Temperature was varied from 4 °C to 37 °C. For post-processing, the 1D-^1^H NMR spectra were processed using Spin Works (version 4.0.0, Copyright ©2013, Kirk Marat, University of Manitoba). All the spectra were referenced to TMS.

The B_1_ strength and pulse duration for LATEST contrast at the horizontal bore 9.4 T was optimized using a 50 mM sample of sodium lactate (pH 7.0) in PBS. Saturation pulses of varying B_1rms_ (1.18 μT to 4.11 μT in steps of 0.59 μT) were acquired from −1.5ppm to +1.5ppm in steps of 0.1 ppm.

CEST imaging was performed using a custom-programmed, segmented radiofrequency GRE readout pulse sequence, with a frequency selective continuous wave saturation preparation pulse. The sequence consists of an 8 s delay, followed by a 5 s saturation pulse, with 780 ms total centric phase encode readout (128 segments, 6.1 ms each). The imaging parameters were: slice thickness = 5 mm, GRE flip angle = 5°, GRE readout TR = 5.6 ms, TE = 2.7 ms, Field of view = 25 × 25 mm^2^, matrix size = 128 × 128, and one saturation pulse at a root mean square B_1_ (B_1rms_) of 100 Hz (2.34 μT). B_0_ correction was done by acquiring WASSR^36^ images at 0.24 μT from −1 to +1 ppm in steps of 0.1 ppm, using the same parameters as CEST. Z-spectra were plotted using the normalized image intensity as a function of the resonance offset of the saturation pulse.

### Phantom Imaging: 7 T

The imaging parameters for 7 T phantom experiments using a 28-channel ^1^H knee coil were: slice thickness = 10 mm, GRE flip angle = 5°, GRE readout TR = 5.6 ms, TE = 2.7 ms, FOV = 130 × 130 mm^2^, SHOT TR = 12 s, matrix size = 128 × 128. A B_1_ titration (1.18 μT to 4.11 μT in steps of 0.59 μT) was performed at three different durations (3 s, 4 s, and 5 s), to determine optimal CEST saturation parameters. The exchange rate was determined by obtaining T_2_ values from lactate phantoms (0, 10, 20, 30, and 50 mM) at 25 °C and from the calculated relaxivity^37^, and using the chemical shift of lactate obtained from the unsuppressed water spectrum at 4 °C (~0.8ppm).

### Lymphoma tumor model: Preparation of lymphoma xenografts

Male athymic nude mice (n = 4) (01B74) 4–6 weeks of age obtained from the National Cancer Institute, Frederick, MD, USA were housed in microisolator cages and had access to water and autoclaved mouse chow *ad libitum*.

The WSU-DLCL2 cell line, diffuse large B-cell lymphoma cells, was kindly provided by Drs Mohammad and Al-Katib (Wayne State University, Detroit, MI, USA). The cells were grown as described^38^ and implanted subcutaneously into the right thigh of 4–6-week-old male athymic nude mice (01B74) 4–6 weeks (National Cancer Institute) by injecting ten million WSU-DLCL2 cells in 0.1mL Hanks’ Balanced Salt Solution (without calcium or magnesium; Invitrogen/Gibco, Carlsbad, CA, USA).

Lymphoma xenografts were allowed to grow until the tumor volume reached ~500 mm^3^. The tumor dimensions were measured with calipers in three orthogonal directions, and the volume was calculated using the equation, V = π(a × b × c)/6, where a, b, and c are the length, width, and depth of the tumor.

### Lymphoma tumor model: Imaging and spectroscopy

The Institutional Animal Care and Use Committees (IACUC) of the University of Pennsylvania approved experimental protocols, and all experiments were carried out in accordance with approved IACUC guidelines. Tumor-bearing mice implanted with lymphoma cells were maintained under 1% isoflurane in 100% oxygen, supplied at 1 L/min. Imaging was performed using a custom-built single frequency (^1^H) slotted tube resonator (inner diameter = 13 mm, outer diameter = 15 mm, depth = 16.5 mm) at a 9.4 T/31 cm horizontal bore Varian system. The animal’s body temperature was maintained at 37 ± 1 °C with the air generated and blowing through a heater (SA Instruments, Inc., Stony Brook, NY). Respiration and body temperature were continuously monitored using a MRI-compatible small animal monitor system (SA Instruments, Inc., Stony Brook, NY). CEST imaging was performed as described for phantoms at 9.4 T with the following sequence parameters: field of view = 25 × 25 mm^2^, slice thickness = 3 mm, flip angle = 15°, TR = 6.2 ms, TE = 2.9 ms, matrix size = 128 × 128. CEST images were collected with B_1rms_ = 1.17 μT for frequencies ranging from −1.5 ppm to +1.5 ppm from bulk water in step size of 0.1 ppm. B_1_ and WASSR B_0_ field maps were also acquired and used to correct the CEST maps as described previously^19^.

Following acquisition of the baseline CEST, sodium pyruvate (300 mM) was delivered through a tail vein catheter (26 Gauge, I.V. Catheters FEP, Tyco Healthcare, Tyco International Ltd., Schaffhausen, Switzerland) at a variable rate using a syringe pump (Harvard Apparatus, Holliston, MA, USA), using the protocol described previously^38^.

Tumor lactate was also measured using HADAMARD SEL-MQC ^1^H-MRS^26^,^39^ in separate experimental sessions. For lactate measurements following pyruvate infusion, tumors were positioned in a home-built, single-frequency (^1^H), slotted-tube resonator (inner diameter, 13 mm; outer diameter, 15 mm; depth, 16.5 mm). A slice-selective double-frequency Hadamard-selective multiple quantum coherence transfer pulse sequence was used to detect lactate and to filter out overlapping lipid signals. The acquisition parameters were as follows: sweep width = 4 kHz; 2048 data points; TR = 8 s; 128 scans. Since there is no published lactate visibility data on this tumor model we have chosen to correlate the lactate peak amplitude values with the LATEST results.

### Human imaging

All human studies were performed under the approved Institutional Review Board (IRB) protocol of the University of Pennsylvania. The studies were carried out in accordance with approved IRB guidelines. Written informed consent was obtained from all subjects after explanation of the study protocol. Lactate CEST imaging was performed on the right calf muscle of 5 healthy male subjects (ages 21–35) on a Siemens 7 T whole-body scanner using a 28-channel ^1^H knee coil. Two baseline CEST images, as well as Water Saturation Shift Reference (WASSR)^36^ B_0_ and B_1_ field maps were acquired pre-exercise, as described previously^19^. WASSR images were collected from −1 to +1 ppm with a step size of 0.05 ppm and saturation pulse train amplitude, (B_1rms_ = 0.29 μT) and 3000-ms duration using the same sequence as used for CEST imaging and identical readout parameters, as described in Kogan *et al.*

Each CEST image was collected using a saturation pulse consisting of a 3 s long saturation pulse of a series of 99.6-ms Hanning windowed saturation pulses (B_1rms_ = 0.73 μT). The parameters for CEST imaging were: slice thickness = 10 mm, SHOT TR = 6 s, TR = 6.1 ms, TE = 2.9 ms, FOV = 140 × 140 mm^2^, matrix size 128 × 128. CEST images were acquired from 0–±0.8 ppm, in steps of 0.1ppm. Plantar flexion exercise was performed in-magnet with a magnetic resonance compatible, pneumatically-controlled foot pedal. The pressure was controlled with a pneumatic gauge and held constant at 9psi for all subjects. The subjects were instructed to exercise until exhaustion, which was approximately 3.5 minutes of intense exercise, with subjects indicating when they could not perform further flexions. Immediately following the exercise, 10 sets of CEST images were acquired with a time resolution of 1.8 minutes. Immediately post-exercise an anatomic image with no saturation was also acquired for muscle segmentation. Additionally, B_1_ and WASSR maps were again acquired at the end of exercise for correction of the post-exercise CEST images.

Lactate spectroscopy was acquired using image-selected *in vivo* spectroscopy^37^ localization followed by a selective multiple quantum coherence (MQC) editing sequence^39^. Spectroscopy was performed on 3 human subjects (ages 24–65), with the same exercise paradigm. A voxel of 30 × 80 × 40 mm was placed over the gastrocnemius muscle. Pre-exercise, spectra were acquired for 96 seconds. Following intense, in-magnet exercise, spectra were acquired for a total of 20 minutes, 48 s. For quantification purposes, both water spectra and lactate spectra were acquired from the same voxel. The details of the lactate-editing spectroscopy sequence can be found in He *et al.*^26^ For these experiments the spectroscopy parameters were: TR = 3 s, TE = 165.6 ms, dummy scans = 4, water averages = 8, lactate edited spectra averages = 8*n, n = 2 for pre-exercise, n = 50 for post-exercise.

### Post processing Methods

CEST asymmetry was calculated using the equation 1,


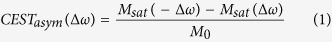


Where M_0_ is the magnetization obtained with saturation at −20ppm, M_sat_ (±Δω) are the magnetizations obtained with saturation at a ‘+’ and ‘−’ offset to the water resonance with a Δω equivalent to the resonance offset of the exchanging spins.

In order to correct for B_0_ inhomogeneities, B_0_ GRE maps were acquired for phantom studies and WASSR maps for *in vivo* studies. Because the –OH protons of lactate resonate ~0.4ppm away from water, an interpolation routine of the z-spectrum from ±1.2ppm with a step-size of 0.005ppm was implemented to correct B_0_ inhomogeneities (see Supplementary Methods).

From the MQC-filtered spectroscopy data, lactate concentration was calculated using the ratio of lactate integrals and water spectra from the same volume, accounting for lactate visibility from double-quantum spectroscopy, and the efficiency factor of the editing (see Supplementary Methods).

## Additional Information

**How to cite this article**: DeBrosse, C. *et al.* Lactate Chemical Exchange Saturation Transfer (LATEST) Imaging *in vivo* A Biomarker for LDH Activity. *Sci. Rep.* **6**, 19517; doi: 10.1038/srep19517 (2016).

## Supplementary Material

Supplementary Information
