# Erratum: Lactate Chemical Exchange Saturation Transfer (LATEST) Imaging *in vivo*: A Biomarker for LDH Activity

**DOI:** 10.1038/srep21813

**Published:** 2016-02-25

**Authors:** Catherine DeBrosse, Ravi Prakash Reddy Nanga, Puneet Bagga, Kavindra Nath, Mohammad Haris, Francesco Marincola, Mitchell D. Schnall, Hari Hariharan, Ravinder Reddy

Scientific Reports 6: Article number: 1951710.1038/srep19517; published online: 01222016; updated: 02252016.

The original version of this Article contained an error in the title of the paper, where “Lactate Chemical Exchange Saturation Transfer (LATEST) Imaging *in vivo*: A Biomarker for LDH Activity” was incorrectly given as “Lactate Chemical Exchange Saturation Transfer (LATEST) Imaging *in vivo* A Biomarker for LDH Activity”. This has now been corrected in the PDF and HTML versions of the Article.

