# Supplementary material for: Lactate Chemical Exchange Saturation Transfer (LATEST) Imaging in vivo A Biomarker for LDH Activity
Source: Sci Rep. 2016 Jan 22;6:19517. doi: 10.1038/srep19517 (PMC4726389; doi:10.1038/srep19517)
Supplement: Supplementary Information [file srep19517-s1.pdf]

Lactate Chemical Exchange Saturation Transfer (LATEST) Imaging in vivo: A Biomarker for LDH Activity

Catherine DeBrosse[1†], Ravi Prakash Reddy Nanga[1†], Puneet Bagga[1], Kavindra Nath[2], Mohammad Haris[3], Francesco Marincola[3], Mitchell D. Schnall[1], Hari Hariharan[1], and Ravinder Reddy[1*]

[1]Center for Magnetic Resonance and Optical Imaging, Department of Radiology, Perelman School of Medicine, University of Pennsylvania, Philadelphia PA
[2] Laboratory of Molecular Imaging, Department of Radiology, Perelman School of Medicine, University of Pennsylvania, Philadelphia PA
[3]Research Branch, Sidra Medical and Research Center, Doha, Qatar
[†]These authors contributed equally to this work

Supplementary Methods

LATEST B_0 correction:

Previous CEST methods have typically been developed for metabolites with resonance frequencies >0.5ppm away from the large bulk water peak. For those metabolites, typically we acquire partial z-spectra for a small set of saturation offset frequencies around +/- CEST offset of interest. Two separate polynomial fits following spline interpolations on the positive and negative frequencies were sufficient for correction of B_0 inhomogeneities[1]. As demonstrated in this work, the hydroxyl proton of lactate resonates ~0.4ppm downfield from water. In this case, a more robust method for B_0 correction is required in post-processing. First an under-sampled raw full z-spectrum (including 0 ppm) is created from each image pixel. Then, a cubic spline interpolation is used to create a finely sampled z-spectrum (x100). Using the B_0 values for each pixel, the z-spectrum is then shifted based on the B_0 value: the spectrum is shifted to the left for negative values, or to the right for positive values. From the shifted spectrum, we can

then calculate the CEST values at the saturation offset of interest (0.4ppm for the lactate hydroxyl proton).

Lactate quantification from ISIS localized Sel-MQC edited spectra:

Lactate concentration was derived from spectroscopy using Sel-MQC filtering, as described in the Methods. In order to quantify the lactate concentration, an ISIS-localized water peak was acquired before the Sel-MQC. The concentration of lactate was obtained using a simple formula:

[lac] = estimated free water conc in muscle * (lactateintegral/waterintegral) * c1;

c1 is a correction factor which is given by the following equation:

c1 = 2.0 * (2 / n) * (1.0/cos(pi*J*t)) * 1/(0.2 * exp(-TE(ms) /144)+0.8* exp(-TE(ms) /37);

where 'n' is the number of spins (n=3 for lactate), 't' is the echo position offset relative to (1/2J) and the previously reported double exponential T_2 decay of lactate rat skeletal muscle[2] , in which 80% of the signal has a T_2 of 37ms, and 20% has a T_2 of 144ms.

References

1. Cai, K. *et al.*, Magnetic resonance imaging of glutamate. *Nat Med*. **18**, 302-6 (2012).

2. Jouvensal, L., Carlier P.G., & Bloch G. Low visibility of lactate in excised rat muscle using double quantum proton spectroscopy. *Magn Reson Med*. **38**, 706-11 (1997).

Supplementary Figures

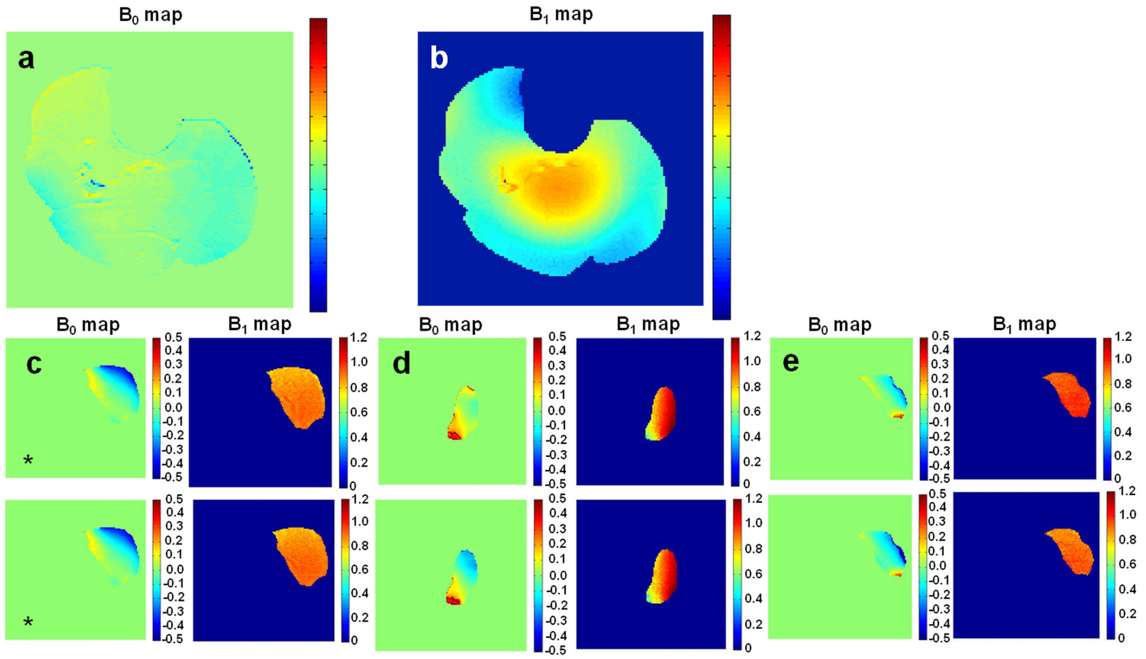

Supplementary Figure 1: B_0 and B_1 correction of *in vivo* calf muscle CEST and lymphoma tumors in mice. (**a**) B_0 (WASSR) map, acquired with B_{1rms} = 0.29µT and 200ms duration, collected from 0 ppm to ±0.5 ppm, in steps of 0.05ppm and (**b**) B_1 GRE map for human calf muscle, (**c*-e, top row**) pre- (**c*-e, bottom row**) and post-infusion B_0 and B_1 maps for three animals with flank tumors.*B_0 map is identical for pre- and post-infusion. No center frequency change, no animal movement.

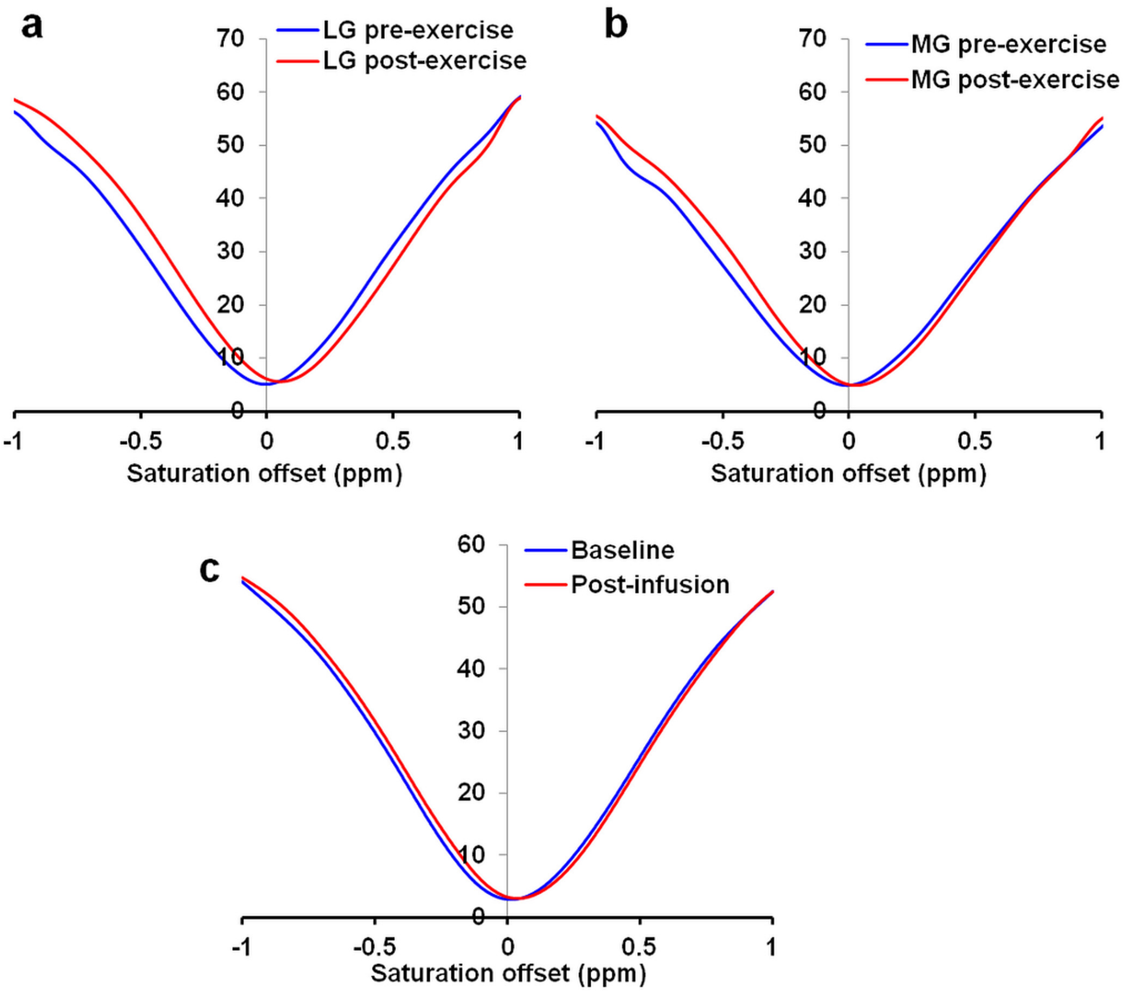

Supplementary Figure 2: Z-spectra for human skeletal muscle and animal tumors. (**a**) Z-spectra for the lateral gastrocnemius muscle region, (**b**) Z-spectra for the medial gastrocnemius muscle region, and (**c**) Z-spectra from the tumor region in a representative animal.

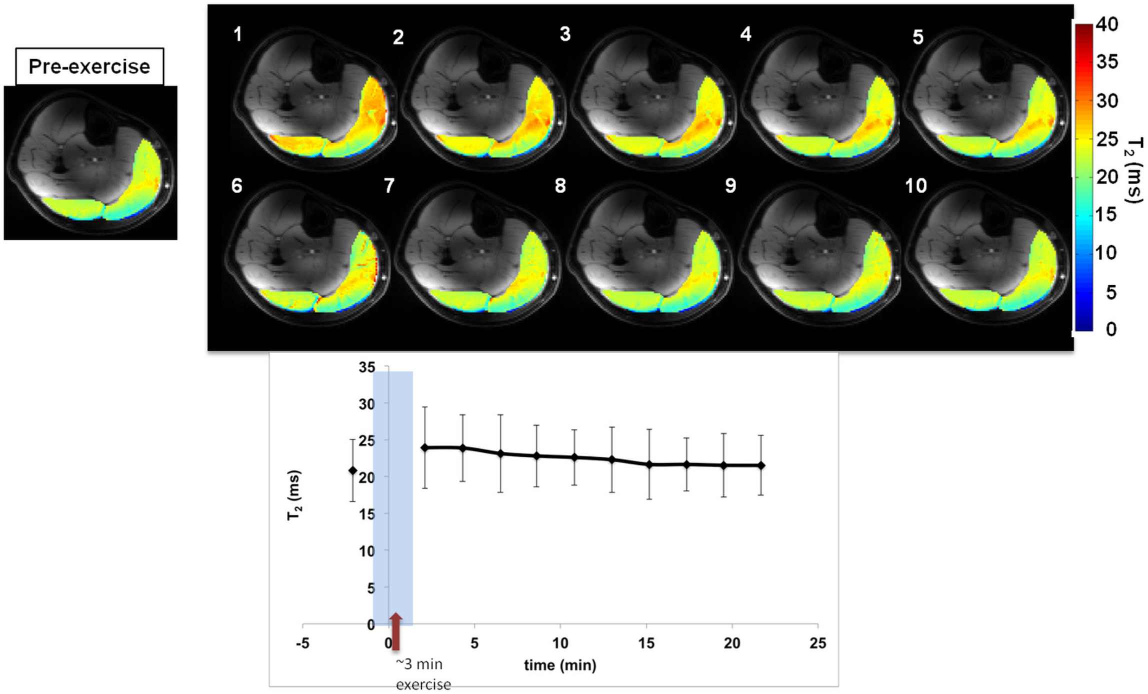

Supplementary Figure 3: Gastrocnemius muscle T_2 measurements. (**a**) T_2 maps overlaid on the anatomical image before exercise, and (**b**) T_2 maps post-exercise, acquired with the same time resolution as the LATEST images, (**c**) T_2 value in ms vs. recovery time in minutes.
